# Hydroxyurea Therapy Mobilises Arachidonic Acid from Inner Cell Membrane Aminophospholipids in Patients with Homozygous Sickle Cell Disease

**DOI:** 10.1155/2011/718014

**Published:** 2011-09-15

**Authors:** A. A. Daak, K. Ghebremeskel, M. I. Elbashir, A. Bakhita, Z. Hassan, M. A. Crawford

**Affiliations:** ^1^Institute of Brain Chemistry and Human Nutrition, Faculty of Life Sciences, London Metropolitan University, London N7 8DB, UK; ^2^Department of Biochemistry, Faculty of Medicine, Khartoum, University of Khartoum, Sudan; ^3^Sickle Cell Referral Clinic, Abnaof Paediatric Hospital, Khartoum, Sudan

## Abstract

The cytotoxic compound hydroxyurea (HU) is effective therapy for sickle cell disease. However, its effect on unsaturated membrane lipids is unknown. Red cell fatty acids were investigated in HU-treated (*n* = 19) and HU-untreated (*n* = 17) sickle cell patients and controls (*n* = 20). The HU-treated compared with the HU-untreated patients had lower arachidonic (AA) acid level in ethanolamine, physphoglycerids (EPG) (22.9 ± 1.2 versus 24.0 ± 1.1%, *P* < 0.05) serine SPG (22.13 ± 2.2 versus 24.9 ± 2.3%, *P* < 0.01) phosphoglycerides. The treated patients and controls had comparable levels of docosahexaenoic (DHA) and total n-3 fatty acids in EPG and choline phosphoglycerides (CPG). In contrast, the untreated group had significantly (*P* < 0.05) lower DHA and total n-3 compared with the controls in EPG (2.7 ± 0.4 versus 3.2 ± 0.6% and 4.6 ± 0.5 versus 5.2 ± 0.7%) and CPG (0.7 ± 0.2 versus 1.0 ± 0.2% and 1.2 ± 0.2 versus 1.4 ± 0.3). HU is known to activate cytosolic phospholipase A2 and cyclooxygenase 2, and from this study, it appears to induce mobilisation of AA from the inner cell membrane EPG and SPG. Hence, eicosanoids generated from the released AA may play a role in clinical improvements which occur in HU-treated patients.

## 1. Introduction

Hydroxyurea (HU) is effective therapy for adults, children and infants with severe sickle cell disease [1–4]. It is thought to reduce the frequency of painful crises by induction of foetal haemoglobin (HbF) and subsequent inhibition of polymerisation of deoxyhaemoglobin S [5]. However, clinical improvements do occur prior to a significant rise in levels of HbF [6]. This suggests that HU may modulate the pathophysiology of the disease by other additional factors. 

HU has been shown to increase red blood cell (RBC) deformability [7], decrease the expression of adhesion molecules on reticulocytes [8], and reduce the translocation of phosphatidylserine from inner to outer leaflet of RBC membrane lipid bilayer [9]. Adhesion, aggregation, and deformability of blood cells are strongly modulated by membrane fatty acids [10–12]. Steady-state sickle cell patients not treated with HU have abnormal RBC, platelets and mononuclear cell fatty acids, which is characterised by an increase in arachidonic acid (AA, 20:4n-6) and a decrease in linoleic acid (LA, 18:2n-6), eicosapentaenoic acid (EPA, 20:5n-3), and docosahexaenoic acid (DHA, 22:6n-3) [13–15]. These findings have led to the postulation that “an imbalance of blood cell membrane n-3 and n-6 fatty acids may be the antecedent of loss of membrane asymmetry, blood cell adhesion, aggregation and, vasoocclusion in SCD” [16]. The aim of this study was to elucidate whether HU treatment modulates RBC membrane fatty acids of steady-state homozygous sickle cell patients (HbSS).

## 2. Subject and Methods

### 2.1. Subjects

Steady-state HbSS sickle cell patients, treated (age 7–26; *n* = 19) and untreated (age 7–22; *n* = 17) with HU, and healthy (HbAA) controls (age 6–21; *n* = 20) matched for ethnicity and economic background were enrolled from the Abnaof Paediatric Hospital, Khartoum, Sudan. The phenotypic characteristic was confirmed by cellulose acetate electrophoresis. The treated patients were on HU for three months or longer. The exclusion criteria were sickle cell crisis, acute illness in the previous month, presence of other chronic diseases, blood transfusion in the previous four months, or pregnancy. The Research Board of the Faculty of Medicine, University of Khartoum, Sudan, approved the study. Self- or investigator-read and explained written consent was obtained from the participants or their guardians. In addition, approval was obtained from the National Research Ethics Service, Southampton & South West Hampshire Research Ethics Committee (A) UK (REC reference: 05/Q1702/48).

After an overnight fast, 5 mL of whole blood was taken from the patients and controls in EDTA tubes. The whole blood was fractionated into RBC and plasma by cold centrifugation at 3000 rpm for 15 min. The top plasma layer was carefully siphoned off and transferred into another tube. The lower red cell layer was washed twice with physiological saline (0.85% NaCl) and centrifuged to remove traces of plasma and buffy coat. The resulting plasma and RBC pellet were stored at −80°C until analysis. 

### 2.2. Analysis of Plasma Lipids

Plasma total cholesterol, high-density lipoprotein cholesterol (HDL-cholesterol), and triglyceride concentrations were analysed by the use of the Ace Alera Clinical Chemistry System (Alfa Wassermann Diagnostic Technologies B.V. Pompmolenlaan 24, 3447 GK Woerden, The Netherlands). Low-density lipoprotein cholesterol (LDL-cholesterol) was computed with the following equation: {LDL-Cholesterol = Total Cholesterol – (HDL-Cholesterol + Triglyceride/5)}.

### 2.3. Analysis of Red Blood Cell Fatty Acids

Total lipids were extracted by the method of Folch et al. [17] by homogenising 0.5 mL of RBC in 2 : 1 v/v chloroform/methanol containing 0.01% (w/v) butylated hydroxytoluene, under nitrogen. The major phospholipid classes were separated by thin-layer chromatography on silica gel plates with the use of the developing solvents: chloroform/methanol/methylamine (65 : 35 : 15 v/v/v). The resulting bands were visualised by spraying the plate with 0.01% (w/v) methanolic 2, 7-dichlorofluorescein and identified by authentic standards developed on the same plates. 

Fatty acid methyl esters (FAMEs) were prepared by heating the phospholipid bands in 4 mL of 15% methanolic acetyl chloride in a sealed vial at 70°C for 3 hours, under nitrogen. The resulting FAMEs were extracted with petroleum spirit, dried, dissolved in heptane, and subsequently separated by a gas chromatograph (HRGC MEGA 2 series, Fisons Instruments, Italy) fitted with a BP 20 capillary column (50 m × 0.32 mm ID, 0.25*:* film). Hydrogen was used as a carrier gas at 2 mL/min, and the injector, oven, and detector temperatures were 250, 230, and 280°C, respectively. 

Quality-certified fatty acid methyl ester standard mixture (Supelco 37 Component FAME Mix. U47885-U, Sigma-Aldrich, Dorset, UK) and GC-MS-authenticated fatty acid methyl esters prepared from lipid extract of vegetable seed oils, which contain alpha-linolenic, gamma-linolenic, and stearidonic acids, and from bovine brain L-A-phosphatidylethanolamine Type 1 (Sigma-Aldrich, Dorset, UK) were used to identify the fatty acids. Peak areas were computed with EZChrom chromatography data system (Scientific Software, Inc, San Ramon, Calif, USA).

### 2.4. Statistical Analysis

The data are expressed as mean ± SD. Unpaired *t*-test was used to explore differences in concentrations of total, HDL, and LDL cholesterol and triglycerides between the HU-treated patients and healthy controls. RBC fatty acids of the HU-treated and untreated patients and healthy controls were compared with one-way ANOVA and the post hoc test for unequal variance, Tamhane's T2. Statistical significance was assumed at a “*P*” value of less than 0.05. The statistical software SPSS for Windows, Version 17 (SPSS Ltd., Woking, Surrey, UK) was used to analyse the data.

## 3. Results

### 3.1. Plasma Lipids

Mean concentrations of total, HDL, and LDL cholesterol and triglyceride of the HU-treated sickle cell patients and healthy controls are presented in Table 2. The patients, compared with their healthy counterparts, had lower total HDL (*P* < 0.001) and LDL (*P* < 0.01) cholesterol.

### 3.2. Red Blood Cell Fatty Acids

Percent fatty acid composition of RBC choline phosphoglycerides (CPG) and sphingomyelin (SPM), the major phospholipids of the outer membrane leaflet, and ethanolamine phosphoglyceride (EPG) and serine (SPG) phosphoglycerides, the dominant phospholipids of the inner membrane leaflet, are shown in Tables 3, 4, 5, and 6, respectively. 

#### 3.2.1. Red Cell CPG

The healthy controls had lower levels of palmitic acid (C16:0), oleic acid (C18:1n-9), total monoenes and adrenic acid (C22:4n-6) (*P* < 0.001) and higher stearic (C18:0) and linoleic (C18:2n-6) acids and total n-6 (*P* < 0.001) fatty acids compared with the HU-treated and untreated patients. In addition, they had higher docosahexaenoic acid (22:6n-3), n-3 metabolites (*P* < 0.01) and total n-3 fatty acids (*P* < 0.05) than the HU-untreated patients. Stearic and gamma linolenic (18:3n-6, GLA) acids (*P* < 0.001) and total saturated fatty acids (*P* < 0.01) were reduced in the HU-untreated compared with the treated patients.

#### 3.2.2. Red Cell SPM

Palmitic, stearic and arachidonic acids and total saturates were elevated (*P* < 0.01), and oleic and nervonic (*P* < 0.01) and total monoenes (*P* < 0.001) were more reduced in the healthy controls than in the HU-untreated patients. The HU-treated compared with the untreated patients had increased palmitic, stearic, and arachidonic acids (*P* < 0.05) and decreased lignoceric acid (C24:0) total monoenes (*P* < 0.001), and nervonic acid (*P* < 0.01).

#### 3.2.3. Red Cell EPG

The healthy control group compared with the HU-treated and untreated patients had reduced stearic, total saturates, adrenic and dihomo gamma linolenic (20:3n-6, DHGLA) (*P* < 0.001) and increased linoleic and eicosapentaenoic (20:5n-3) acids (*P* < 0.001). The HU-treated patients had lower arachidonic, adrenic, osbond (22:5n-6) (*P* < 0.05), and n-6 metabolites and total n-6 (*P* < 0.001) than their untreated counterparts.

#### 3.2.4. Red Cell SPG

Both the HU-treated and untreated patients had lower linoleic (*P* < 0.001) and higher DHGLA, adrenic acid, osbond acid and total n-6 (*P* < 0.01) compared with the healthy control subjects. The HU-treated patients had lower GLA, eicosadienoic (20:2n-6), arachidonic acids, and osbond acid, n-6 metabolites and total n-6 (*P* < 0.01) than their untreated counterparts.

## 4. Discussion

The reduced concentrations of total, HDL, and LDL cholesterol in the HU-treated sickle cell patients are consistent with the low level of HDL, LDL [18–22], and total cholesterol [23] reported in HU-untreated steady-state sickle cell patients. The patients and healthy control subjects in the current study were broadly matched for age, gender (Table 1), ethnicity, and socioeconomic background. Consequently, the observed reduction in cholesterol levels in the HU-treated patients was unlikely to have been a reflection of genetic or environment factors. It has been postulated hypocholesterolemia in sickle cell patients is due to haemodilution, down regulation of cholesterol synthesis, and decreased transfer of cholesterol from membrane to circulating high-density lipoprotein (HDL) because of reduced activity of lecithin-cholesterol acyltransferase [23, 24]. In the current study, the HU patients and healthy controls had a comparable level of triacylglycerols. Hence, the hypocholesterolemia in the patients could not be explained by haemodilution. Likewise, downregulation of biosynthesis of cholesterol may not be a factor as there is evidence that RBCs of sickle cell patients have higher content of cholesterol [25, 26]. SCD patients have lower level of apolipoprotein A-I [25, 27, 28], and deoxygenated HbS red cells have been shown to have increased uptake of cholesterol analogue [29]. Hence, it is plausible; the hypocholesterolemia observed in the patients was a reflection of increased uptake by RBC membranes and reduced transfer to circulating HDL. 

The current comparative study of Sudanese homozygous sickle cell patients and healthy controls revealed both HU-treated and untreated patients had abnormal red cell fatty acid pattern, which was more pronounced in the latter group. The remarkable anomalies in the HU-untreated patients compared with the healthy controls were decreased linoleic and stearic and increased palmitic and oleic acids in CPG, decreased palmitic and stearic and increased nervonic and lignoceric acids in SPM, decreased linoleic acid, EPA, and DHA and increased stearic, adrenic, and osbond acids in EPG and decreased linoleic and oleic and increased adrenic and osbond acids. These findings are broadly consistent with the previous findings in serum phospholipids [30], red cell total phospholipids [31], red cell phospholipid classes [13, 15], and total lipids of mononuclear cells and platelets [16]. These findings in steady-state homozygous sickle cell patients of different ethnic, cultural, and socioeconomic backgrounds demonstrate the disease induces blood cell membrane fatty acid perturbation. This could be due to (a) metabolic dysfunction-impaired synthesis, uptake, and/or enhanced turnover; (b) peroxidation caused by iron overload. Both the HU-untreated patients and healthy controls had very low n-3 fatty acids compared with the British and Nigerian subjects previously investigated by our group [15]. This difference is most likely a reflection of the n-3 fatty acid status of the populations studied since we have observed North Sudanese maternal milk [32] and blood (unpublished) have very low content of these nutrients. 

Hydroxyurea had a profound effect on fatty acid composition of membrane phospholipids, particularly sphingomyelin, and ethanolamine and serine phosphoglycerides. The striking effect on the former phospholipid was a reduction of 18.1, 26.7, and 20.6% of behenic (C20:0), lignoceric (C22:0), and nervonic (C24:1n-9) acids, respectively. The HU-induced changes in sphingomyelin composition are significant since lipid rafts, the highly ordered membrane microdomains, which are thought to play a pivotal role in membrane trafficking, signal transduction, and gene and protein expression, are rich in sphingomyelin [33, 34]. Moreover, saturated and mono-unsaturated fatty acids are structural components and major functional determinants of sphingomyelin. 

In addition, hydroxyurea reduced arachidonic acid (AA, C20:4n-6) significantly in both ethanolamine (−4.7%) and serine (−11.1%) phosphoglycerides, phospholipids found predominately in the inner leaflet of membrane lipid bilayer. Such reductions were not apparent in choline phosphoglycerides and sphingomyelin, which are principally found in the outer leaflet of membrane lipid bilayer. Hydroxyurea generates nitric oxide *in vivo* [35–37] and *in vitro *[38, 39], and the functionally coupled cPLA_2_ *α* and cyclooxygenase 2 (COX2) [40] are activated by nitric oxide [41, 42].

Prostaglandin E2 (PGE2), which is a metabolite of AA and a vasodilator, has been shown to induce the synthesis of foetal haemoglobin in peripheral blood derived from erythroid colonies from normal and sickle cell adults [43]. In rats, the HU-induced synthesis of foetal haemoglobin is obviated by aspirin, the potent inhibitor of COX [44]. Hence, it is tenable to suggest, in the HU-treated patients, AA is selectively released by the activation of the arachidonic-acid-selective cytosolic phospholipase A_2_ *α* (cPLA_2_ *α*) and subsequently metabolised by COX2 to generate PGE2. 

Consistent with our previous findings in HU-untreated HbSS patients [16], the HU-untreated patients in this study had significantly lower levels of DHA and total n-3 fatty acids in CPG and EPG and of EPA in EPG, SPG, and SPM compared with healthy controls. In contrast, in the HU-treated patients, the reduction in n-3 fatty acids was restricted only to EPA in EPG. It appears the n-3 fatty acid abnormality often observed in HU-untreated patients was partially ameliorated by hydroxyurea treatment. This modulation of membrane fatty acid composition would be expected to help enhance transmembrane ion flux, cell hydration, rheology, and deformability [45–47], factors which are known to improve in HU-treated sickle cell patients [7, 48, 49].

This investigation demonstrates, hydroxyurea modulates red blood cell membrane fatty acid abnormalities including the n-3/n-6 imbalance reported previously in steady-state homozygous sickle cell patients. These modulations in synergy with the HU-generated vasodilators, nitric oxide and PGE2, maybe play a critical role in clinical improvements which occur prior to the increased synthesis of HbF in treated patients.

The current study did not investigate the effect of hydroxyurea on metabolism of eicosanoids derived from arachidonic acid, oxidative stress, and fatty acid composition of other blood cells, such as platelets and leukocytes. These limitations are potential lines of inquiry which may need to be explored in future studies.

## Figures and Tables

**Table 1:** Characteristics of the HU-treated and untreated HbSS patients and healthy (HbAA) subjects.

Characteristics	HU-treated HbSS	HU-untreated HbSS	Healthy control (HbAA)
Number of subjects	19	17	20
Gender (Male/Female)	8/11	7/10	9/11
Age ± SD (year)	14.5 ± 4.3	14.5 ± 6.1	9.7 ± 6.7
Weight ± SD (kg)	36 ± 7.1	31.5 ± 11.8	36.1 ± 14.6
Height ± SD (cm)	146.9 ± 15.3	127.8 ± 25.5	146.3 ± 15.0

**Table 2:** Plasma lipid concentrations of steady state HU treated HbSS sickle cell patients and HbAA healthy controls.

Plasma lipids	HbAA (*n* = 20)	HbSS (*n* = 17)
LDL-cholesterol (mmol/L)	2.4 ± 0.5	1.7 ± 0.7**
HDL-cholesterol (mmol/L)	1.1 ± 0.3	0.8 ± 0.2***
Total cholesterol (mmol/L)	3.71 ± 0.56	2.7 ± 0.8***
Triacylglycerol (mmol/L)	1.01 ± 0.4	1.24 ± 0.03

HbSS patients versus HbAA controls: **P < 0.01, ***P < 0.001;

**Table 3:** Fatty acid composition of red blood cell choline phosphoglycerides of HU-untreated (*n* = 17) and treated (*n* = 19) HbSS patients and HbAA controls (*n* = 20).

Fatty acids	HbSS (untreated)	HbSS (treated)	HbAA (healthy control)
16:0 (Palmitic acid)	36.7 ± 1.6	36.3 ± 1.7^×××^	31.8 ± 2.5^+++^
18:0 (Stearic acid)	8.9 ± 0.7***	10.3 ± 1.2^×××^	13.2 ± 1.0^+++^
20:0 (Arachidic acid)	0.1 ± 0.03***	0.2 ± 0.1^×^	0.1 ± 0.1
22:0 (Behenic acid)	tr.	tr.	tr.
24:0 (Lignoceric acid)	0.04 ± 0.01	0.05 ± 0.01	.04 ± 0.01
*∑*saturates	46.0 ± 1.3**	47.2 ± 0.8^××^	45.2 ± 1.9

16:1n-7 (Palmitoleic acid)	0.3 ± 0.1	0.3 ± 0.1	0.3 ± 0.1
18:1n-7 (Vaccenic acid)	1.6 ± 0.2	1.7 ± 0.3^×^	1.4 ± 0.2^+^
18:1n-9 (Oleic acid)	18.3 ± 1.1	17.7 ± 1.5^×××^	14.2 ± 1.5^+++^
24:1n-9 (Nervonic acid)	tr.	tr.	tr.
*∑*monoenes	20.2 ± 1.1	19.7 ± 1.6^×××^	15.9 ± 1.6^+++^

18:2n-6 (Linoleic acid)	16.3 ± 1.4	15.4 ± 2.6^×××^	20.7 ± 2.1^+++^
18:3n-6 (*γ*-linolenic acid)	0.1 ± 0.02***	0.1 ± 0.03	0.1 ± 0.05
20:2n-6 (Eicosadienoic acid)	0.5 ± 0.1	0.4 ± 0.2	0.5 ± 0.1
20:3n-6 (Dihomo-*γ*-linolenic)	1.9 ± 0.4	1.7 ± 0.2^××^	2.1 ± 0.1
20:4n-6 (Arachidonic acid)	9.6 ± 0.7	9.5 ± 1.1	9.4 ± 1.5
22:4n-6 (Adrenic acid)	0.9 ± 0.2	0.9 ± 0.2^×××^	0.7 ± 0.1^+++^
22:5n-6 (Osbond acid)	0.5 ± 0.1	0.5 ± 0.1^×^	0.6 ± 0.2^+^
*∑*metabolites	13.6 ± 0.7	13.1 ± 1.3	13.4 ± 1.7
*∑*n-6	29.9 ± 1.6	28.4 ± 1.9^×××^	34.1 ± 2.7

18:3n-3 (*α*-Linolenic acid)	tr.	tr.	tr.
20:5n-3 (Timnodonic acid)	0.1 ± 0.02	0.1 ± 0.05	0.11 ± 0.03
22:5n-3 (Clupanodonic acid)	0.3 ± 0.06	0.3 ± 0.07	0.3 ± 0.05
22:6n-3 (Cervonic acid)	0.7 ± 0.2	0.8 ± 0.4	1.0 ± 0.2^++^
*∑*metabolites	1.1 ± 0.2	1.2 ± 0.4	1.3 ± 0.3^++^
*∑*n-3	1.2 ± 0.2	1.2 ± 0.4	1.4 ± 0.3^+^

N6/N3	26.58 ± 5.03	24.91 ± 6.78	25.65 ± 5.09

tr.: trace.

HU-untreated versus treated HbSS patients, *P < 0.05, **P < 0.01, ***P < 0.001.

HU-untreated HbSS patients versus HbAA healthy controls, ^+^ *P* < 0.05, ^++^ *P* < 0.01, ^+++^ *P* < 0.001.

HU-treated HbSS patients versus HbAA healthy controls, ^ ×^ *P* < 0.05, ^ ××^ *P* < 0.01, ^×××^ *P* < 0.001.

**Table 4:** Fatty acid composition of red blood cell sphingomyelin of HU-untreated (*n* = 17) and treated (*n* = 19) HbSS patients and HbAA healthy controls (*n* = 20).

Fatty acids	HbSS (untreated)	HbSS (treated)	HbAA (healthy control)
16:0	21.1 ± 4.3*	25.3 ± 3.5	26.1 ± 2.2^++^
18:0	8.7 ± 2.2*	11.9 ± 3.5	11.3 ± 2.0^++^
20:0	2.0 ± 0.2	1.00 ± 0.2^×××^	1.44 ± 0.2^+++^
22:0	8.1 ± 1.5*	6.6 ± 1.3	7.6 ± 1.1
24:0	25.3 ± 4.8***	18.5 ± 2.9^××^	21.6 ± 2.6^+^
*∑*saturates	63.9 ± 3.8	63.8 ± 3.1^×××^	68.2 ± 1.4^++^

16:1n-7	tr.	tr.	tr.
18:1n-7	0.3 ± 0.1***	0.7 ± 0.1^××^	0.5 ± 0.1^+++^
18:1n-9	3.8 ± 1.9	3.4 ± 0.6^×××^	2.0 ± 0.3^++^
24:1n-9	17.8 ± 3.6**	14.2 ± 1.7	13.9 ± 1.7^++^
*∑*monoenes	21.9 ± 2.8***	18.3 ± 2.0^×^	16.5 ± 1.8^+++^

18:2n-6	2.1 ± 1.1	2.3 ± 0.6	2.0 ± 0.3
18:3n-6	tr.	tr.	tr.
20:2n-6	0.4 ± 0.3**	0.1 ± 0.04^××^	0.09 ± 0.04^++^
20:3n-6	0.3 ± 0.1**	0.5 ± 0.1^××^	0.3 ± 0.1
20:4n-6	3.7 ± 1.3*	4.9 ± 1	5.1 ± 1.3^++^
22:4n-6	1.8 ± 1.0	2.0 ± 0.8^×^	1.3 ± 0.4
22:5n-6	1.4 ± 0.6	1.3 ± 0.6	1.1 ± 0.3
*∑*metabolites	7.5 ± 2.9	8.4 ± 1.9	7.9 ± 2.1
*∑*n-6	9.6 ± 3.5	10.8 ± 1.7	9.9 ± 2.2

18:3n-3	tr.	tr.	tr.
20:5n-3	0.11 ± 0.02***	0.07 ± 0.02	0.06 ± 0.02^+++^
22:5n-3	0.4 ± 0.2	0.45 ± 0.2	0.4 ± 0.1
22:6n-3	1.4 ± 0.7	1.8 ± 0.8	1.7 ± 0.6
*∑*metabolites	1.9 ± 0.8	2.4 ± 0.9	2.1 ± 0.7
*∑*n-3	1.9 ± 0.8	2.4 ± 0.9	2.1 ± 0.7

N6/N3	5.3 ± 1.6	5.3 ± 1.7	4.9 ± 1.1

tr.: trace.

HU-untreated versus treated HbSS patients: *P < 0.05, **P < 0.01, ***P < 0.001.

HU-untreated HbSS patients versus HbAA healthy controls: ^+^ *P* < 0.05, ^++^ *P* < 0.01, ^+++^ *P* < 0.001.

HU-treated HbSS patients versus HbAA healthy controls: ^×^ *P* < 0.05, ^××^ *P* < 0.01, ^×××^ *P* < 0.001.

**Table 5:** Fatty acid composition of red blood cell ethanolamine phosphoglycerides of HU-untreated (*n* = 17) and treated (*n* = 19) HbSS sickle cell patients and HbAA healthy controls (*n* = 20).

Fatty acids	HbSS (untreated)	HbSS (treated)	HbAA (healthy control)
16:0	12.8 ± 1.3	12.8 ± 1.3	12.8 ± 1.2
18:0	7.9 ± 0.9	8.7 ± 1.4^×××^	6.0 ± 0.7^+++^
20:0	0.1 ± 0.02	0.08 ± 0.03	0.06 ± 0.03
22:0	0.03 ± 0.01***	0.07 ± 0.02	0.08 ± 0.04^+++^
24:0	0.06 ± 0.03*	0.08 ± 0.02^×××^	0.06 ± 0.02
*∑*saturates	21.02 ± 1.0	21.9 ± 1.5^×××^	19.1 ± 1.1^+++^

16:1n-7	0.1 ± 0.03***	0.1 ± 0.02	0.1 ± 0.04^+++^
18:1n-7	0.9 ± 0.2	1.2 ± 0.8	0.9 ± 0.1
18:1n-9	13.1 ± 1.2	12.1 ± 1.1	12.9 ± 0.7
24:1n-9	tr.	tr.	tr.
*∑*monoenes	14.0 ± 1.3	13.2 ± 1.1	13.9 ± 1.0

18:2n-6	4.9 ± 0.7	4.9 ± 0.9^×××^	6.7 ± 0.9^+++^
18:3n-6	0.2 ± 0.05***	0.04 ± 0.01	0.04 ± 0.01^+++^
20:2n-6	0.6 ± 0.2	0.5 ± 0.1	0.5 ± 0.1
20:3n-6	1.4 ± 0.3	1.3 ± 0.2^×××^	1.0 ± 0.1^+++^
20:4n-6	24.0 ± 1.1*	22.9 ± 1.2^××^	24.8 ± 1.5
22:4n-6	10.8 ± 0.1*	9.89 ± 1.0^×××^	8.6 ± 0.7^+++^
22:5n-6	2.6 ± 0.4*	2.2 ± 0.5	1.9 ± 0.3^+++^
*∑*metabolites	39.4 ± 1.3***	36.8 ± 2.2	36.8 ± 1.7^+++^
*∑*n-6	44.3 ± 1.5***	41.4 ± 2.5^×^	43.9 ± 1.8

18:3n-3	0.08 ± 0.04	0.08 ± 0.02	0.1 ± 0.03
20:5n-3	0.11 ± 0.03*	0.15 ± 0.05^×××^	0.23 ± 0.06^+++^
22:5n-3	1.7 ± 0.3	1.6 ± 0.3	1.7 ± 0.2
22:6n-3	2.7 ± 0.4	2.9 ± 0.88	3.2 ± 0.6^+^
*∑*metabolites	4.5 ± 0.5	4.6 ± 1.1	5.1 ± 0.7^+^
*∑*n-3	4.6 ± 0.5	4.7 ± 1.1	5.2 ± 0.7^+^

N6/N3	9.8 ± 1.4	9.3 ± 2.34	8.5 ± 1.3^+^

tr.: trace.

HU-untreated versus treated HbSS patients: *P < 0.05, **P < 0.01, ***P < 0.001.

HU-untreated HbSS patients versus HbAA healthy controls: **^+^** *P* < 0.05, **^++^** *P* < 0.01, **^+++^** *P* < 0.001.

HU-treated versus HbAA healthy controls: ^×^ *P* < 0.05, ^××^ *P* < 0.01, ^×××^ *P* < 0.001.

**Table 6:** Fatty acid composition of red blood cell serine phosphoglycerides of HU-untreated (*n* = 17) and treated (*n* = 19) HbSS sickle cell patients and HbAA healthy controls (*n* = 20).

Fatty acids	HbSS (untreated)	HbSS (treated)	HbAA (healthy control)
16:0	4.4 ± 0.9	5.5 ± 1.6	5.2 ± 1.2
18:0	41.9 ± 1.3	42.9 ± 1.9	41.6 ± 1.1
20:0	0.3 ± 0.1	0.3 ± 0.06^××^	0.2 ± 0.07^++^
22:0	0.1 ± 0.04***	0.2 ± 0.1	0.2 ± 0.07
24:0	tr.	tr.	tr.
*∑*saturates	46.9 ± 0.9***	49.2 ± 0.8^×××^	47.5 ± 1.0

16:1n-7	tr.	tr.	tr.
18:1n-7	0.4 ± 0.9***	0.8 ± 0.2	0.7 ± 0.1^+++^
18:1n-9	4.9 ± 1.03*	6.0 ± 1.23	5.1 ± 1.0
24:1n-9	0.03 ± 0.01***	0.4 ± 0.31	0.3 ± 0.23^+++^
*∑*monoenes	5.4 ± 1.1**	7.1 ± 1.5^××^	5.4 ± 1.2

18:2n-6	3.20 ± 0.6	3.3 ± 0.6^×××^	6.1 ± 1.6^+++^
18:3n-6	0.2 ± 0.06***	0.06 ± 0.03^×××^	0.02 ± 0.02^+++^
20:2n-6	0.5 ± 0.1***	0.2 ± 0.04	0.3 ± 0.09^+++^
20:3n-6	3.2 ± 0.8	2.9 ± 0.8^×××^	2.1 ± 0.3^+++^
20:4n-6	24.9 ± 2.3**	22.13 ± 2.2^×××^	25.7 ± 1.9
22:4n-6	6.7 ± 1.1	6.1 ± 1.0^×××^	3.9 ± 0.8^+++^
22:5n-6	3.4 ± 0.7**	2.7 ± 0.58^××^	2.1 ± 0.5^+++^
*∑*metabolites	38.8 ± 1.4***	34.1 ± 1.9	33.9 ± 2.4^+++^
*∑*n-6	42.0 ± 1.2***	37.0 ± 2.0^×××^	40.1 ± 2.7^++^

18:3n-3	tr.	tr.	tr.
20:5n-3	0.07 ± 0.01	0.08 ± 0.09	0.11 ± 0.04^++^
22:5n-3	1.4 ± 0.3	1.2 ± 0.3	1.1 ± 0.2^++^
22:6n-3	3.0 ± 0.6	2.8 ± 0.8	2.7 ± 0.8
*∑*metabolites	4.4 ± 0.7	4.1 ± 1.0	3.9 ± 1.1
*∑*n-3	4.4 ± 0.7	4.1 ± 1.03	3.9 ± 0.1

N6/N3	9.7 ± 1.9	9.7 ± 2.6	11.0 ± 2.9

tr.: trace.

HU-untreated versus treated HbSS patients: *P < 0.05, **P < 0.01, ***P < 0.001.

HU-untreated HbSS patients versus HbAA healthy controls: ^+^ *P* < 0.05, ^++^ *P* < 0.01, ^+++^ *P* < 0.001.

HU-treated HbSS Patients versus HbAA healthy controls: ^×^ *P* < 0.05, ^××^ *P* < 0.01, ^×××^ *P* < 0.001.
